# Immediate allergic hypersensitivity reactions (anaphylactic shock) to intramuscular dexamethasone in an asthmatic child: a case report

**DOI:** 10.1186/s13256-025-05433-6

**Published:** 2025-10-13

**Authors:** Rashid Mahrous, Wafaa Mahrous, M. Mahrous

**Affiliations:** 1 Faculty of Medicine, Menoufia University, Menoufia, Egypt; 2 Faculty of Pharmacy, Tanta University, Tanta, Egypt; 3 National Liver Institute, Menoufia University, Menoufia, Egypt

**Keywords:** Allergic hypersensitivity, Dexamethasone, Asthma, IgE-mediated reaction, Anaphylactic shock, Glucocorticoids, Desensitization, Case report

## Abstract

**Introduction:**

Glucocorticoids are essential in treating various conditions due to their immunosuppressive, anti-inflammatory, and antiallergenic properties. However, they can cause hypersensitivity reactions that may go undiagnosed. Recognizing corticosteroid hypersensitivity is crucial, as demonstrated by a case in which a patient with asthma experienced severe anaphylaxis after receiving dexamethasone.

**Case presentation:**

A 5-year-old Egyptian male with asthma was admitted with severe symptoms, including shortness of breath, low oxygen saturation, and tachycardia. After initial treatment with nebulized medications, he was given intramuscular dexamethasone but quickly developed severe anaphylaxis, characterized by rash, angioedema, and anaphylactic shock. Immediate treatment with epinephrine, oxygen (intubation), saline, and cetirizine was administered, and the patient stabilized after a second dose of epinephrine. Following 24 hours of observation, his condition improved, and we discussed safe alternatives with a clinical pharmacist.

**Conclusion:**

Steroid-induced allergic reactions, although rare, can lead to life-threatening conditions such as anaphylaxis, as illustrated in this case.

## Introduction

Glucocorticoids are prescribed for their immunosuppressive, antiproliferative, anti-inflammatory, and antiallergenic effects and are integral to the management of numerous conditions, including malignancies, transplantation, autoimmune and allergic diseases, and asthma. They are also administered to prevent late-phase anaphylactic reactions [1].

However, corticosteroids can induce immediate and delayed hypersensitivity reactions. Although these reactions are not common, hypersensitivity to corticosteroids can often go undiagnosed because symptoms can mimic some of the emergency conditions for which they are given, such as shock, status asthmaticus, or anaphylaxis [2].

Patients are therefore at risk of receiving the same offending corticosteroid more than once, which might be life-threatening [3].

Due to the wide usage of corticosteroid medications, it is clinically important for corticosteroid hypersensitivity reactions to be considered [4].

We present the case of a patient with a history of asthma, who suffered an episode of serious anaphylaxis after intramuscular administration of dexamethasone.

## Case presentation

A 5-year-old Egyptian male child, weighing 18 kg, who had a history of mild intermittent asthma controlled with as-needed use of an albuterol inhaler only and did not have any previous history of using inhaled steroids, was admitted to our facility with an acute exacerbation of asthma. His symptoms included shortness of breath, use of accessory breathing muscles, chest retractions, and low oxygen saturation around 89%. He also presented with tachycardia and a respiratory rate of approximately 25 breaths per minute.

Management was initiated with nebulized high-dose albuterol and ipratropium, which were administered every 15 minutes for three doses. The patient showed slight improvement and was then given dexamethasone at a dose of 0.6 mg/kg intramuscularly.

However, within a few minutes after receiving dexamethasone, the patient developed a skin rash, hives, itching, altered mental status, angioedema, cyanosis of the lips and extremities, and swelling of the tongue with severe wheezing. His condition rapidly deteriorated with a sudden drop in blood pressure below a mean of 50 mmHg and oxygen saturation below 80%, indicating anaphylactic shock.

Immediate management included the administration of 0.2 mg intramuscular epinephrine, the application of a nonrebreather mask with 15 L of oxygen, a bolus of 400 mL of saline, and 2.5 mg cetirizine intravenously. Initially, the patient’s airway was intubated, and his lungs were ventilated with an Ambu bag delivering 15 L of oxygen. The patient improved after the second dose of epinephrine. We followed up with him for 24 hour. All vital signs and symptoms improved. Extubation was performed when the patient no longer needed the assistance of the tube for breathing. The diagnosis of an allergic reaction to dexamethasone was confirmed by a skin prick test, and we systematically excluded potential co-triggers, including food intake, insect stings, and drug exposures, to minimize confounding factors. We reported the case in the Incident Reporting System and discussed safe alternatives with a clinical pharmacist.

## Discussion

Steroid-induced allergic reactions are rare. According to Klein-Gitelman *et al.* and Borja *et al.* [5, 6], the overall prevalence of type I immunoglobulin E (IgE)-mediated steroid hypersensitivity includes immediate hypersensitivity reactions in 0.1–0.3% of patients under systemic corticosteroid treatment. However, the true prevalence of such reactions is unknown, as large control studies are unavailable and most relevant data are based on case reports [4]. Delayed hypersensitivity reactions, more common than immediate reactions, were reported in 0.5–5% of patients under corticosteroid treatment, especially topical treatment [7, 8].

The attribution of allergic reactions to corticosteroids was not clearly established until 1974, when Mendelson *et al*. reported the first immediate anaphylactic reaction following the intravenous administration of hydrocortisone and methylprednisolone succinate in an asthmatic patient. It was shown that the allergic reactions experienced (urticaria, angioedema, severe bronchospasm) were due to steroid molecules, not excipients [4, 9].

Immediate reactions usually begin within 1 hour of administration, whereas delayed reactions start after 1 hour. This classification system aims to distinguish IgE-mediated reactions, which are mostly immediate, from other types. It is crucial to identify IgE-mediated reactions because they carry the risk of anaphylaxis upon re-exposure [10, 11].

Several patients have been reported to have allergic reactions to carboxymethyl cellulose and, less frequently, macrogol (polyethylene glycol [PEG]). Some injectable glucocorticoids contain PEGs or polysorbates (e.g., methylprednisolone acetate contains PEG 3350, and triamcinolone acetonide contains polysorbate 80). However, in our case, the patient received dexamethasone, which contains neither PEG nor polysorbate 80 [12].

For skin testing, preservative-free glucocorticoid solutions are preferred whenever possible. If a preservative-free preparation is unavailable, and/or if a patient had an allergic reaction after receiving a glucocorticoid preparation containing a preservative, then a positive result to the preservative-containing preparation should be followed by skin testing with a preparation of the preservative (prick-puncture and, if negative, intradermal) [13].

Despite using dexamethasone, consider Class C corticosteroids such as methylprednisolone to be a less allergenic alternative for patients with allergies to methylprednisolone and hydrocortisone. However, it is essential to consider the risk of hypersensitivity and anaphylaxis, as seen in our case [14].

In patients without an alternative safe corticosteroid agent or when a challenge is avoided due to a history of severe corticosteroid-induced anaphylaxis, desensitization to the culprit corticosteroid can be considered. This procedure should be performed under close clinical observation and is only temporarily effective [15, 16]. However, repeated desensitization each time steroid therapy is required may be neither practical nor safe. Instead, noninvasive *in vitro* testing, such as the basophil activation test (BAT), should be considered to confirm the allergy. In addition, testing safer alternatives, such as inhaled corticosteroids, can help reduce the need for repeated provocation testing and minimize the risk of future severe reactions [17, 18].

## Conclusion

Steroid-induced allergic reactions, although rare, can lead to life-threatening conditions such as anaphylaxis. Clinicians should be aware of this possibility and its potential life-threatening effects. Continued research and reporting of such cases are essential to better understand the prevalence, mechanisms, and management strategies of steroid-induced allergic reactions, ensuring safer therapeutic outcomes for patients.

## Data Availability

The datasets used and/or analyzed during the current study are available from the corresponding author on reasonable request.

## Abbreviations

IgE: Immunoglobulin E
PEG: Polyethylene glycol
SOB: Shortness of breath
BAT: Basophil activation test
